# Growth differentiation factor 15 (GDF15) is associated with non-alcoholic fatty liver disease (NAFLD) in youth with overweight or obesity

**DOI:** 10.1038/s41387-022-00187-2

**Published:** 2022-02-22

**Authors:** Brittany Galuppo, Cristiana Agazzi, Bridget Pierpont, Jennifer Chick, Zhongyao Li, Sonia Caprio, Nicola Santoro

**Affiliations:** 1 Department of Pediatrics, Yale School of Medicine, New Haven, CT USA; 2 Department of Medicine and Health Sciences, “V. Tiberio,” University of Molise, Campobasso, Italy

**Keywords:** Obesity, Metabolic syndrome

## Abstract

**Objective:**

Growth differentiation factor 15 (GDF15) has been associated with food intake and weight regulation in response to metabolic stress. In animal models, it has been noted that it may play a role in the progression of non-alcoholic fatty liver disease (NAFLD), the leading cause of chronic liver disease in children.

**Design:**

In the current study, we explored the association of circulating plasma concentrations of GDF15 with NAFLD in youth with overweight/obesity, and whether changes in plasma concentrations in GDF15 parallel the changes in intrahepatic fat content (HFF%) over time.

**Methods:**

Plasma GDF15 concentrations were measured by ELISA in 175 youth with overweight/obesity who underwent an oral glucose tolerance test (OGTT) and magnetic resonance imaging (MRI) to assess intrahepatic, visceral, and subcutaneous fat. Baseline fasting GDF15 concentrations were measured in twenty-two overweight/obese youth who progressed (*n* = 11) or regressed (*n* = 11) in HFF% by more than 30% of original over a 2-year period.

**Results:**

Youth with NAFLD had significantly higher plasma concentrations of GDF15 than those without NAFLD, independent of age, sex, ethnicity, BMI *z*-score (BMIz), and visceral fat (*P* = 0.002). During the OGTT, there was a decline in plasma GDF15 concentrations from 0 to 60 min, but GDF15 concentrations returned to basal levels by the end of the study. There was a statistically significant association between change in HFF% and change in GDF15 (*P* = 0.008; *r*^2^ = 0.288) over ~2 years of follow-up.

**Conclusions:**

These data suggest that plasma GDF15 concentrations change with change in intrahepatic fat content in youth with overweight/obesity and may serve as a biomarker for NAFLD in children.

## Introduction

Non-alcoholic fatty liver disease (NAFLD) is the most common liver disease in pediatrics and affects ~30% of children and adolescents with obesity in the United States [1–4]. Despite the pervasiveness and severity of the disease, little is known about its pathogenesis and biomarkers associated with its onset and development. Recently, metabolic research has focused on growth differentiation factor 15 (GDF15), a stress-induced cytokine belonging to the transforming growth factor beta superfamily [5]. As of today, GDF15 has been associated with many conditions including cardiac dysfunction, metformin response, and tumor-related cachexia [6–8]. Metabolic effects of GDF15 are mediated by GDNF family receptor α–like (GFRAL), an endogenous receptor, uniquely expressed at the level of the central nervous tissue [9]. GDF15, however, is up-regulated in almost any cell or tissue in response to stress, thus suggesting its key role as an anti-inflammatory cytokine [10, 11].

Recent studies have shown that GDF15 is associated with NAFLD in adults. In particular, GDF15 represents an independent determinant of fibrosis severity in NAFLD: (1) patients with more severe chronic liver diseases have proportionately higher GDF15 values and (2) plasma GDF15 concentrations are associated with liver fibrosis regardless of diabetes status [12, 13].

So far, there are no studies investigating whether plasma GDF15 concentrations in youth with obesity are associated with NAFLD. In this study, we sought to determine whether GDF15 plasma concentrations are associated with (1) intrahepatic fat content, (2) changes in intrahepatic fat accumulation over time, and (3) the metabolic phenotype related to NAFLD in youth with obesity. To achieve our aims, we measured GDF15 in 175 youth with overweight/obesity who underwent an oral glucose tolerance test and an MRI to measure intrahepatic fat content. Moreover, we analyzed changes in GDF15 over time in 22 youth who were followed-up for ~2 years and also showed changes (increase or decrease) in intrahepatic fat content of at least 30%. Herein, we show that in youth with overweight/obesity, GDF15 plasma concentration is associated with NAFLD and NAFLD-related phenotypes and that changes in plasma GDF15 concentration are driven by changes in intrahepatic fat content.

## Subjects and methods

### Study population

A cross-sectional cohort of children and adolescents with overweight/obesity was identified from the Yale Pediatric Obesity Clinic main research study cohort (New Haven, CT). A multiethnic group of 175 youth with overweight/obesity between the ages of 8 and 21 years was selected based on protocol inclusion and exclusion criteria. Per the study protocol, participants had a BMI ≥ 85^th^ percentile according to U.S. Centers for Disease Control and Prevention (CDC) growth charts [14], and were excluded for the use of medication on a chronic basis, presence of a chronic condition, baseline blood creatinine >1.0 mg/dL, pregnancy, presence of endocrinopathies, or use of metformin. Subjects were otherwise healthy children and adolescents.

Baseline fasting plasma GDF15 concentrations were measured in all subjects and in a subgroup of subjects with (*n* = 10) and without (*n* = 10) NAFLD, matched for age, sex, BMIz, and ethnicity, during an OGTT. Longitudinal analyses were performed in a subgroup of 22 youth with overweight/obesity who were followed up for ~2 years (1.79 years ± 0.178) and either increased (*n* = 11) or decreased (*n* = 11) HFF% by more than 30% in the two-year period. All subjects underwent an oral glucose tolerance test and abdominal MRI to measure intrahepatic fat content (HFF%), visceral, and subcutaneous fat as previously described [2, 15]. The study was approved by the Yale University Human Investigations Committee in accordance with the Helsinki Declaration of 1975 as revised in 1983. Written consent was obtained from each patient after full explanation of the purpose and nature of all procedures used.

### Biochemical analyses

Plasma GDF15 concentrations were measured using Human Quantikine ELISA kits (SGD150, R&D Systems Inc., Minneapolis, MN, USA) according to manufacturer’s instructions. The detection threshold for this kit is 4.39 pg/mL and the assay range is 23.4–1,500 pg/mL. Plasma glucose was measured at bedside during the OGTT using the YSI2700-STAT-Analyzer (Yellow Springs Instruments, Yellow Springs, OH, USA), plasma insulin was measured by antibody radioimmunoassay from Millipore Sigma (Billerica, MA, USA), and lipid levels were measured using Auto-Analyzer (model 747-200, Roche Diagnostics, Indianapolis, IN, USA).

### Statistical analyses

Categorical data are presented as counts (percent) and were analyzed using a *χ*^2^ test. Continuous variables are presented as mean (standard deviation) or as median (interquartile range) for HFF%. Non-normally distributed variables were log-transformed to approximate normality, except for HFF% that was transformed into rank (PROC RANK). A Mann-Whitney test was used to compare the differences between subjects with and without NAFLD and to compare differences between the cross-sectional cohort and the subgroup for longitudinal follow-up. A general linear model (PROC GLM) was used to test differences between NAFLD groups, and age, sex, ethnicity, BMIz, and visceral adipose tissue (VAT) were used as covariates. A GLM was also used to assess the association of GDF15 with VAT, using age, sex, ethnicity, BMIz, and HFF% as covariates. A simple regression analysis was used to test the association between change in intrahepatic fat content and change in GDF15 concentration. Statistical significance was established at an alpha of 0.05. Analyses were performed using SAS 9.4 (Cary, NC) and GraphPad Prism software 9.0.0 (San Diego, CA).

## Results

### GDF15 in Youth with and without NAFLD

**Fig. 1:**
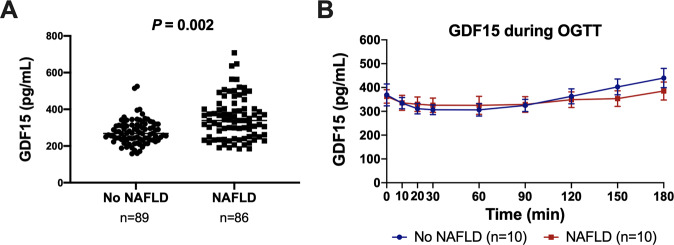
GDF15 in a cross-sectional cohort of youth with and without NAFLD. **A** Fasting plasma GDF15 concentration in overweight/obese youth with or without NAFLD. *P*-value is from Mann–Whitney test. **B** Plasma GDF15 levels during an OGTT in overweight/obese youth with and without NAFLD, matched for age, sex, ethnicity, BMIz. Youth without NAFLD are depicted in blue and youth with NAFLD are depicted in red. Differences in GDF15 concentrations during an OGTT between the groups were not statistically significant. Error bars represent standard error of the mean.

In Table 1, the clinical characteristics of study participants stratified by presence or absence of NAFLD, as defined by HFF% greater than or equal to 5.5%, are shown. Of the 175 youth with overweight/obesity included in the study, 89 youth did not have NAFLD and 86 youth demonstrated the MRI features of NAFLD. There were no statistically significant differences between the two groups in terms of age (*P* = 0.224) and sex (*P* = 0.174). BMI *z*-score, measures of glucose metabolism and insulin sensitivity, and ALT levels were higher in the group with NAFLD. Fasting plasma GDF15 levels were significantly different between the two groups, independent from age, sex, BMI *z*-score, ethnicity, and VAT (*P* = 0.002) (Fig. 1A). No association was found between GDF15 levels and BMIz (*P* = 0.085) (Supplemental Fig. 1).

**Table 1:** The clinical characteristics of the cross-sectional cohort grouped by presence or absence of NAFLD are shown.

	Youth without NAFLD (*n* = 89)	Youth with NAFLD (*n* = 86)	*P*-value
Age (y)	13.1 ± 3.09	13.6 ± 3.03	0.224
Sex (M/F)	(27/62)	(42/44)	0.174
Ethnicity (C/AA/H/O)	21/35/29/4	17/8/54/7	*<1.0* *×* *10*^*−10*^
BMIz	2.23 ± 0.461	2.37 ± 0.367	0.145
Fasting glucose (mg/dL)	90 ± 7	92 ± 8	*0.039*
Fasting insulin (mU/L)	31 ± 24	41 ± 26	*2.32* *×* *10*^*−4*^
2-h glucose (mg/dL)	119 ± 28	129 ± 32	*0.029*
WBISI	2.25 ± 1.14	1.56 ± 0.895	*3.65* *×* *10*^*−5*^
Triglycerides (mg/dL)	103 ± 43.8	170 ± 145	*3.75* *×* *10*^*−4*^
HDL (mg/dL)	43.1 ± 8.48	40.5 ± 10.5	*0.044*
LDL (mg/dL)	93.8 ± 27.5	97.9 ± 24.5	0.361
Total cholesterol (mg/dL)	161 ± 30.5	169 ± 40.7	0.316
ALT (IU/L)	21.7 ± 25.3	60.1 ± 74.3	*<1.0* *×* *10*^*−10*^
HFF %	1.92 ± 1.71	16.9 ± 10.4	*<1.0* *×* *10*^*−10*^
VAT (cm^2^)	64.3 ± 27.5	91.1 ± 36.9	*5.84* *×* *10*^*−6*^
SAT (cm^2^)	514 ± 194	519 ± 186	0.966
VAT/VAT+SAT	0.114 ± 0.038	0.153 ± 0.047	*2.22* *×* *10*^*−7*^

A Mann–Whitney test was used to compare the differences between subjects with and without NAFLD. Glucose, insulin, triglycerides, HDL, LDL, total cholesterol, and ALT were measured from plasma. Data are shown as mean ± standard deviation.

*AA* African American, *C* Caucasian, *F* female, *H* Hispanic, *M* male, *O* Other, *WBISI* whole body insulin sensitivity index.

Italics values identify statistical significance.

GDF15 was associated with VAT independent of age, sex, ethnicity, and BMIz (*P* = 0.008), but after adjustment for intrahepatic fat content, the association became marginally significant (*P* = 0.042).

Similarly, there was also an association between WBISI and GDF-15 independent from age, sex, ethnicity, and BMIz (*p* = 0.006), which disappeared after adjusting for HFF% (*P* = 0.082).

We measured plasma GDF15 during a 3-h OGTT and observed a slight reduction in GDF15 concentrations from 0 to 60 min. The GDF15 concentrations tended to have a greater initial reduction (0–60 min) and later rebound (60–180 min) in the group without NAFLD compared to the group with NAFLD (Fig. 1B). The delta change in GDF15 concentration from baseline (0 min) to the nadir (60 min) was similar between the groups with (81.2 ± 39.0) and without (58.5 ± 17.7) NAFLD (*P* = 0.971).

### Changes in GDF15 over time

**Fig. 2:**
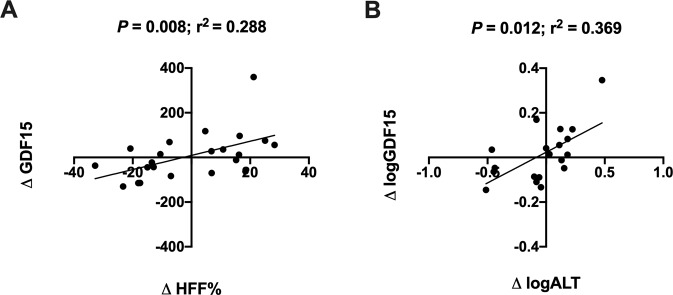
Change in GDF15 in youth that increased or decreased in HFF% by at least 30% of original in a two-year period. **A** Correlation between change in HFF% and change in plasma GDF15 concentrations. **B** Correlation between change in logALT concentrations and change in logGDF15 concentrations. *P*-values and *r*^2^ values are from Spearman rank correlation.

A subgroup of 22 youth with obesity who increased or decreased in HFF% by at least 30% of original over a two-year period was assessed for changes in plasma GDF15 concentrations. The subgroup did not differ significantly from the cross-sectional cohort in terms of age, sex, and BMIz, but differed significantly in measures of glucose and insulin metabolism, as most subjects in the subgroup had NAFLD to begin with. In the subgroup, there was an association between delta change in HFF% and delta change in plasma GDF15 concentrations, in which the change in GDF15 paralleled the change in HFF% (*P* = 0.008; *r*^2^ = 0.288) (Fig. 2A). Similar observations were made for change in plasma GDF15 concentrations and changes in plasma ALT concentrations (*P* = 0.012; *r*^2^ = 0.369) (Fig. 2B).

## Discussion

In the present study, we observed an association between plasma concentrations of GDF15 and NAFLD and an association between changes in HFF% and changes in plasma GDF15 concentrations. Specifically, in subjects who experienced a reduction of NAFLD severity, plasma concentrations of GDF15 decreased, while, in those in whom the disease progressed, plasma concentrations of GDF15 increased significantly. Consistent with what has been shown before in adults, GDF15 concentrations in plasma tend to decrease during an OGTT and return to the initial concentrations by the end of the 3-hour study [16]. Other studies in adults have shown that GDF15 concentration tends to increase as the NAFLD phenotype worsens. Koo et al. [12] observed in a group of 190 adults with NAFLD, that GDF15 concentrations were higher in subjects with a greater degree of fibrosis. In our study, the observation that the degree of change in GDF15 paralleled the degree of change in intrahepatic fat content over time corroborates the hypothesis that GDF15 synthesis might increase with an increase of lipid synthesis in the liver. In a study that aimed to explore the role of GDF15 in the modulation of immune response, Luan et al. [17] showed that GDF15 concentrations are increased with increased production of lipids in the liver of a mouse model. In the study, the authors demonstrated that GDF15 may provide a protective effect against the inflammatory insult induced by viruses and bacteria and concluded that GDF15 attenuates the inflammatory response by modulating lipid metabolism [17].

It is likely that the upregulation of GDF15 might be a protective mechanism against the inflammatory response induced by free fatty acids in the context of fatty liver disease and the inflammation sustained by macrophages that leads to NASH and fibrosis. Moreover, Luan et al. [17] also showed that GDF15 infusion enhances hepatic triglycerides export into the circulation. If this is confirmed in humans, it may be another mechanism through which GDF15 may prevent liver damage in individuals with NAFLD. The hypotheses were generated by the findings from Luan et al. [17] seem to support the findings by Kim et al. [18], showing that GDF15 downregulates the expression of genes involved in the development of liver fibrosis and that the overexpression of GDF15 in transgenic mice alleviates hepatic inflammation and the NASH phenotype [18]. Furthermore, additional studies demonstrated that GDF15 expression in the liver can significantly promote beta-oxidation of fatty acids and ketogenesis in hepatocytes, thus playing a protective role against hepatic steatosis and inflammation [19, 20].

We acknowledge that our study has some limitations. The main limitation of the study is that, although the association observed between GDF15 and hepatic fat is strong, this study does not include information about liver histology that would be needed to understand whether GDF15 is related to changes in liver histology such as inflammation and fibrosis. Moreover, we are not able to explain whether this association underlies a causal relationship, or that it is merely an epiphenomenon within the context of more complex metabolic changes. Therefore, larger longitudinal and interventional studies are warranted to shed light on the metabolic dynamics linking GDF15 and NAFLD in youth with overweight or obesity.

## Supplementary information

Supplemental Figure 1
