# Supplementary material for: Growth differentiation factor 15 (GDF15) is associated with non-alcoholic fatty liver disease (NAFLD) in youth with overweight or obesity
Source: Nutr Diabetes. 2022 Feb 22;12:9. doi: 10.1038/s41387-022-00187-2 (PMC8863897; doi:10.1038/s41387-022-00187-2)
Supplement: Supplementary file 1 — Supplemental Figure 1 [file 41387_2022_187_MOESM1_ESM.docx]

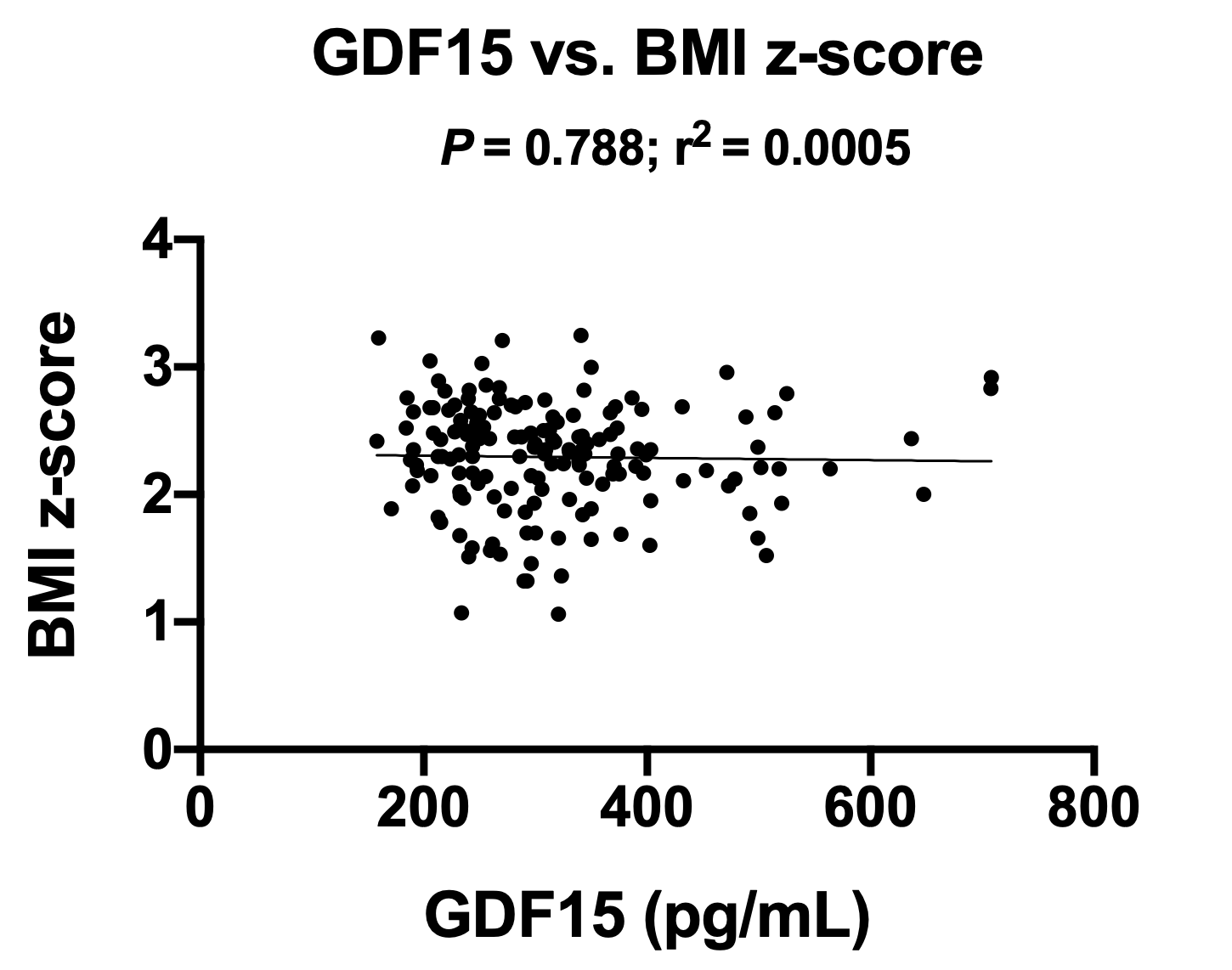


**Supplemental Figure 1.** Correlation between fasting plasma GDF15 concentration and BMI z-score. P-values and r^2^ values are from Spearman rank correlation.
